# Integrated long-term safety of 10-year ozanimod treatment: results from clinical trials in patients with moderate-to-severe ulcerative colitis or relapsing multiple sclerosis

**DOI:** 10.1093/ibd/izaf319

**Published:** 2026-02-12

**Authors:** David T Rubin, Silvio Danese, Hiroshi Nakase, Ryan C Ungaro, Douglas C Wolf, Olga Alekseeva, AnnKatrin Petersen, Zhaohui Liu, Dimpy Mehra, Anjali Jain, Mark T Osterman, Anthony Krakovich, Jon V Riolo, Erik DeBoer, James Appio, Preetika Sinh, Bruce A C Cree, Jeffrey A Cohen, Peter Irving

**Affiliations:** University of Chicago Medicine Inflammatory Bowel Disease Center, Chicago, IL, United States; Department of Gastroenterology and Endoscopy, IRCCS Ospedale San Raffaele and University Vita-Salute San Raffaele, Milan, Italy; Sapporo Medical University, Sapporo, Japan; Icahn School of Medicine at Mount Sinai, New York, NY, United States; Center for Crohn’s Disease & Ulcerative Colitis, Atlanta Gastroenterology Associates, Atlanta, GA, United States; Department of Gastroenterology, Nizhny Novgorod Regional Clinical Hospital, Nizhny Novgorod, Russia; Bristol Myers Squibb, Princeton, NJ, United States; Bristol Myers Squibb, Princeton, NJ, United States; Bristol Myers Squibb, Princeton, NJ, United States; Bristol Myers Squibb, Princeton, NJ, United States; Bristol Myers Squibb, Princeton, NJ, United States; Bristol Myers Squibb, Princeton, NJ, United States; Bristol Myers Squibb, Princeton, NJ, United States; Bristol Myers Squibb, Princeton, NJ, United States; Bristol Myers Squibb, Princeton, NJ, United States; Division of Gastroenterology, Medical College of Wisconsin, Milwaukee, WI, United States; Department of Neurology, Weill Institute for Neurosciences, University of California San Francisco, San Francisco, CA, United States; Mellen Center for Multiple Sclerosis Treatment and Research, Cleveland Clinic, Cleveland, OH, United States; Guy’s and St Thomas’ NHS Foundation Trust, London, United Kingdom

**Keywords:** ozanimod, ulcerative colitis, relapsing multiple sclerosis

## Abstract

**Background:**

Ozanimod is a once-daily oral selective sphingosine 1-phosphate receptor modulator approved for the treatment of moderately to severely active ulcerative colitis (UC) or relapsing multiple sclerosis (RMS). Previous analyses in both indications demonstrated favorable long-term safety profiles of ozanimod. Here we report an integrated analysis of the long-term safety of ozanimod in patients with UC or RMS.

**Methods:**

Data were pooled in patients with UC who received ozanimod in phase 2, phase 3, and open-label extension (OLE) trials and in patients with RMS who received ozanimod in an OLE trial after completing any phase 1-3 parent trial. Safety assessments included treatment-emergent adverse events (TEAEs) and laboratory abnormalities.

**Results:**

Overall, 3652 patients with UC or RMS had 16 144 patient-years (PY) of ozanimod exposure over 10 years of follow-up. The most common TEAEs were nasopharyngitis, headache, and coronavirus disease 2019. Rates of TEAEs leading to treatment discontinuation (1.4/100 PY) and TEAEs of special interest, including serious infections (1.0/100 PY), herpes zoster (0.5/100 PY), malignancies (0.4/100 PY), bradycardia (0.1/100 PY), sinus bradycardia (0.04/100 PY), complete atrioventricular block (0.01/100 PY), and macular edema (0.1/100 PY), were low. No serious hepatic events or Hy’s law cases occurred. Absolute lymphocyte count of < 200 cells/µL was not temporally associated with serious or opportunistic infections.

**Conclusions:**

Long-term exposure to ozanimod is well tolerated in patients with moderate to severe UC or RMS, confirming the previously established safety profile of ozanimod.

**Clinical Trial Registry:**

NCT01647516; NCT02435992; NCT02576717

Key Messages *What is already known?*

Ozanimod was well tolerated for up to 3 years in patients with ulcerative colitis (UC) who received continuous ozanimod through the True North trial and its open-label extension (OLE) and for up to 7 years in patients with relapsing multiple sclerosis (RMS) in the DAYBREAK OLE trial.

*What is new here?*

This integrated analysis of ozanimod UC and RMS clinical trials confirms the established safety profile of ozanimod with >16 000 PY of exposure and up to 10 years of follow-up.

*How can this study help patient care?*

The findings of this integrated analysis may increase provider confidence in the generally consistent long-term safety profile of ozanimod across disease states and populations.

## Introduction

Ozanimod is a once-daily oral selective sphingosine 1-phosphate (S1P) receptor modulator approved in multiple countries for the treatment of adults with moderately to severely active ulcerative colitis (UC) or relapsing multiple sclerosis (RMS).^1^^,^^2^ ­Ozanimod binds with S1P receptors 1 and 5 but has no activity against S1P receptors 2–4.^3^ This lack of interaction with S1P receptors 3 and 4 may contribute to differences in safety profiles between ozanimod and less selective S1P receptor modulators, such as fingolimod, which binds all S1P receptors except S1P receptor 2.^4^^,^^5^ In a matching-adjusted indirect comparison in patients with RMS, ozanimod appeared to have a more favorable benefit–risk profile than fingolimod.^6^ During first-dose monitoring in clinical trials, ozanimod was associated with a significantly lower risk of cardiac events, and there was a lesser need for extended first-dose monitoring with ozanimod than fingolimod. Further, ozanimod was associated with a significantly lower risk of adverse events (AEs), AEs leading to treatment discontinuation, herpetic infections, basal cell carcinoma, bradycardia, and hepatic enzyme elevations than fingolimod after 2 years of treatment. Results from a more recent matching-adjusted indirect comparison in patients with RMS were similar, with ozanimod being associated with a lower risk of AEs and AEs leading to treatment discontinuation than fingolimod after 2 years of treatment.^7^

The long-term safety of ozanimod was previously characterized in patients with UC with 434 patient-years (PY) of exposure for up to 3 years of continuous treatment in the phase 3 True North parent trial and its open-label extension (OLE) trial (up to OLE week 94) in a subset of OLE patients and in patients with RMS with 12 665 PY of exposure for up to 7 years of continuous treatment in the phase 3 DAYBREAK OLE trial (15 556 PY and up to 10 years of exposure in the parent trials and DAYBREAK).^8^ Overall, long-term treatment with ozanimod was well tolerated in patients with UC or RMS. Treatment-emergent adverse events (TEAEs) were manageable and infrequently led to treatment discontinuation. Rates of TEAEs of special interest based on prior associations with S1P receptor modulation, including serious and opportunistic infections, malignancies, macular edema, and cardiac events (eg, bradycardia), were generally low.

Together, these data from ozanimod clinical trials demonstrated a favorable safety profile, but additional UC and RMS clinical trial data have become available since these previous analyses, and integrated analyses of safety data across the UC and RMS indications have not previously been performed.^8^^,^^9^ An integrated analysis allows for a better understanding of the overall safety profile of ozanimod in a larger database across diverse disease states and patient populations that may be at differing risks of AEs. The objective of this analysis was to provide an integrated assessment of the safety of ozanimod in patients with moderate to severe UC or RMS with additional ozanimod exposure in clinical trials.

## Materials and methods

### Clinical trials

Data were analyzed from clinical trials of ozanimod in patients with moderately to severely active UC or RMS. The UC clinical trials included the phase 2 TOUCHSTONE parent (December 26, 2012–March 10, 2015) and TOUCHSTONE OLE (May 17, 2013–August 29, 2019) trials (NCT01647516) and the phase 3 True North parent (NCT02435992; August 12, 2015–March 27, 2020) and True North OLE (NCT02531126; December 2, 2015–ongoing [at the time of this analysis]) trials.^8^^,^^10^^,^^11^ Details of the designs of these trials are shown in Figure S1 and have also been previously reported.^8^^,^^10–12^

One RMS trial was included, the phase 3 DAYBREAK OLE trial (NCT02576717; October 16, 2015–January 5, 2023), which enrolled patients who completed any phase 1-3 parent trial.^9^ Details of the design of this trial are shown in Figure S2 and have also been previously reported.

All trials were conducted in accordance with Good Clinical Practice guidelines and the Declaration of Helsinki principles and complied with applicable national, state, and local regulatory requirements. Protocols were reviewed and approved by the institutional review board at each trial site. All patients provided written informed consent.

### Patients

Data are presented separately in the UC and RMS populations and in the pooled UC + RMS population. The UC population included patients treated with ozanimod 0.92 mg pooled from the phase 2 TOUCHSTONE trial, the phase 3 True North trial, and the respective OLE trials. Data in patients from all trials were examined from a start date of December 2, 2015, through an interim data cutoff of November 17, 2023 (up to OLE Week 190), as the True North OLE was ongoing at the time of this analysis. Some patients could have begun ozanimod treatment prior to the start date of this analysis, as all trials but the True North OLE started before December 2, 2015. Patients who received placebo in TOUCHSTONE or True North were not included in this analysis.

The RMS population included patients treated with ozanimod 0.92 mg in the phase 3 DAYBREAK OLE trial after completing any phase 1-3 parent trial. Data in patients from the DAYBREAK OLE trial were examined from the beginning of the trial (October 16, 2015) through the last patient assessment (January 5, 2023; the database lock was April 7, 2023).

### Safety assessments

TEAEs, serious TEAEs, TEAEs leading to treatment discontinuation, and TEAEs of special interest were assessed during the entire analysis period. TEAEs were considered serious if they were fatal or life-threatening, required inpatient hospitalization or prolongation of existing hospitalization, resulted in persistent or significant disability/incapacity, or were congenital abnormalities/birth defects; important medical events that required medical intervention to prevent one of these outcomes were also considered serious TEAEs. TEAE severity was defined as follows: mild TEAEs are transient and do not interfere with the patient’s daily activities; moderate TEAEs introduce a low level of inconvenience or concern to the patient and may interfere with daily activities; and severe TEAEs are incapacitating and interrupt the patient’s usual daily activities. TEAEs of special interest based on prior associations with S1P receptor modulation have historically included infections, malignancies, cardiovascular disorders, macular edema, and hepatic effects. Of note, there was a difference in conduct of the UC vs RMS trials, such that UC disease flares were reported as TEAEs in the UC trials, whereas RMS disease flares were not captured as TEAEs in the RMS trials. Exposure-adjusted incidence rates (EAIRs) per 100 PY were calculated for all TEAEs to adjust for time on trial. Laboratory abnormalities were also assessed and included absolute lymphocyte count (ALC) reductions and hepatic enzyme elevations that occurred during the OLE trials only.

## Results

### Patient disposition and characteristics

The pooled UC + RMS population included 3652 patients: 1158 with UC and 2494 with RMS. At data cutoff (November 17, 2023), the RMS DAYBREAK OLE trial had been completed, and 9.5% of the patients with UC were still participating in the True North OLE (Table 1).

**Table 1:** Patient disposition.

Patient data	UC (*n* = 1158; PY^a^ = 3479)	RMS (*n* = 2494; PY^a^ = 12 665)	UC + RMS (*n* = 3652; PY^a^ = 16 144)
**Exposure, years, mean (SD)**	3.0 (2.4)	5.1 (1.5)	4.4 (2.1)
**Range**	0.0–10.1	0.0–6.8	0.0–10.1
**Patients ongoing in OLE at data cutoff, *n* (%)**	110 (9.5)	0	110 (3.0)
**Primary reason for withdrawal from the OLE, *n* (%)**			
**Withdrawal by patient**	166 (14.3)	259 (10.4)	425 (11.6)
**Lack of efficacy**	201 (17.4)	72 (2.9)	273 (7.5)
**AE**	61 (5.3)	84 (3.4)	145 (4.0)
**Other**	91 (7.9)	50 (2.0)	141 (3.9)
**Physician decision**	41 (3.5)	20 (0.8)	61 (1.7)
**Lost to follow-up**	0	38 (1.5)	38 (1.0)
**Protocol deviation**	7 (0.6)	9 (0.4)	16 (0.4)
**Noncompliance with trial drug**	13 (1.1)	0	13 (0.4)
**Death**	0^b^	11 (0.4)^c^	11 (0.3)
**Pregnancy**	7 (0.6)	0	7 (0.2)
**Trial termination by sponsor**	0	1 (0.04)	1 (0.03)

Abbreviations: AE, adverse event; OLE, open-label extension; PY, patient-years; RMS, relapsing multiple sclerosis; UC, ulcerative colitis.

a PY were calculated as the sum of the number of years on trial contributed by each patient from the time of first dose to last date on trial.

b Five patients died after discontinuing for other reasons: 4 AEs and 1 withdrawal.

c A total of 15 patients died, of whom 4 died after discontinuing for other reasons: 1 voluntarily withdrawal, 1 AE, 1 physician decision, and 1 death during the safety follow-up after completing the trial.

The total pooled UC + RMS population represented 16 144 PY of ozanimod exposure, with a mean duration of 4.4 years (standard deviation [SD] 2.1; range 0–10.1). The UC population had 3479 PY of ozanimod exposure, with a mean duration of 3.0 years (SD, 2.4; range, 0–10.0). The RMS population had 12 665 PY of ozanimod exposure, with a mean duration of 5.1 years (SD, 1.5; range, 0–6.8).

Baseline patient characteristics are shown in Table 2. The mean age was 41.6 years in patients with UC and 36.1 years in patients with RMS; 40.6% of the UC population and 66.9% of the RMS population were female. The mean age of all patients in the combined UC + RMS population was 37.8 years; 58.5% were female.

**Table 2:** Baseline patient characteristics.

Characteristics	UC (*n* = 1158)	RMS (*n* = 2494)	UC + RMS (*n* = 3652)
**Age, years, mean (SD)^a^**	41.6 (13.3)	36.1 (9.2)	37.8 (11.0)
**Age categories, years, *n* (%)**			
** 18–29**	262 (22.6)	664 (26.6)	926 (25.4)
** 30–39**	302 (26.1)	915 (36.7)	1217 (33.3)
** 40–49**	253 (21.8)	703 (28.2)	956 (26.2)
** 50–59**	200 (17.3)	212 (8.5)	412 (11.3)
** 60–69**	127 (11.0)	0	127 (3.5)
** 70–75**	14 (1.2)	0	14 (0.4)
**Sex, *n* (%)**			
** Female**	470 (40.6)	1668 (66.9)	2138 (58.5)
** Male**	688 (59.4)	826 (33.1)	1514 (41.5)
**Race, *n* (%)**			
** White**	1036 (89.5)	2474 (99.2)	3510 (96.1)
** Asian**	68 (5.9)	3 (0.1)	71 (1.9)
**Black or African American**	31 (2.7)	14 (0.6)	45 (1.2)
** Other**	22 (1.9)	3 (0.1)	25 (0.7)
** Not reported**	1 (0.1)	0	1 (0.03)
**Body mass index, kg/m^2^, mean (SD)**	25.4 (5.4)	24.3 (4.8)	24.7 (5.0)

Abbreviations: RMS, relapsing multiple sclerosis; UC, ulcerative colitis.

a Age at parent trial baseline.

### Overall safety

EAIRs of TEAEs, serious TEAEs, and TEAEs leading to treatment discontinuation were higher in patients with UC than patients with RMS (Table 3). The most frequently occurring TEAEs were lymphopenia, COVID-19, and anemia in the UC population and nasopharyngitis, headache, and COVID-19 in the RMS and pooled UC + RMS populations. Some TEAEs, including anemia, arthralgia, and those related to lymphocyte decreases or hepatic enzyme elevations, occurred more frequently in patients with UC than those with RMS. Other TEAEs, including headache, back pain, depression, and some infections, occurred more frequently in patients with RMS than those with UC. The most frequently occurring serious TEAEs and TEAEs leading to treatment discontinuation are shown in Table S1 and Table S2, respectively.

**Table 3:** Overall safety in the UC, RMS, and pooled UC + RMS populations.

TEAEs	UC (*n* = 1158)		RMS (*n* = 2494)		UC + RMS (*n* = 3652)	
	*n* (%)	EAIR/100 PY^a^	*n* (%)	EAIR/100 PY^a^	*n* (%)	EAIR/100 PY^a^
**Any TEAE**	884 (76.3)	81.1	2219 (89.0)	65.1	3103 (85.0)	69.0
**Serious TEAEs**	215 (18.6)	7.0	381 (15.3)	3.2	596 (16.3)	4.0
**TEAEs leading to treatment discontinuation**	119 (10.3)	3.5	98 (3.9)	0.8	217 (5.9)	1.4
**Most frequent TEAEs (≥5% in any group)**						
** Nasopharyngitis**	98 (8.5)	3.0	531 (21.3)	5.0	629 (17.2)	4.5
** Headache**	79 (6.8)	2.4	426 (17.1)	3.8	505 (13.8)	3.5
** COVID-19**	118 (10.2)	3.6	412 (16.5)	3.4	530 (14.5)	3.4
** Lymphopenia^b^**	151 (13.0)	4.9	257 (10.3)	2.2	408 (11.2)	2.8
**Upper respiratory tract infection**	65 (5.6)	2.0	310 (12.4)	2.7	375 (10.3)	2.5
**Lymphocyte count decreased^b^**	98 (8.5)	3.0	235 (9.4)	2.0	333 (9.1)	2.2
** Hypertension^c^**	75 (6.5)	2.3	230 (9.2)	1.9	305 (8.4)	2.0
** Back pain**	44 (3.8)	1.3	239 (9.6)	2.0	283 (7.7)	1.9
** GGT increased^b^**	73 (6.3)	2.2	200 (8.0)	1.7	273 (7.5)	1.8
** Arthralgia**	79 (6.8)	2.4	161 (6.5)	1.3	240 (6.6)	1.6
** ALT increased^b^**	94 (8.1)	3.0	126 (5.1)	1.0	220 (6.0)	1.4
** Anemia^b^**	101 (8.7)	3.1	103 (4.1)	0.8	204 (5.6)	1.3
** Urinary tract infection^d^**	23 (2.0)	0.7	169 (6.8)	1.4	192 (5.3)	1.2
** Bronchitis**	31 (2.7)	0.9	157 (6.3)	1.3	188 (5.1)	1.2
** Respiratory tract infection**	17 (1.5)	0.5	165 (6.6)	1.4	182 (5.0)	1.2
**Viral respiratory tract infection**	23 (2.0)	0.7	145 (5.8)	1.2	168 (4.6)	1.1
** Depression**	12 (1.0)	0.4	127 (5.1)	1.0	139 (3.8)	0.9
** UC**	69 (6.0)	2.0	2 (<1)^e^	0.02	71 (1.9)	0.4

Abbreviations: ALT, alanine aminotransferase; EAIR, exposure-adjusted incidence rate; GGT, gamma-glutamyl transferase; PY, patient-years; RMS, relapsing multiple sclerosis; TEAE, treatment-emergent adverse event; UC, ulcerative colitis.

a PY were calculated as the sum of the number of years on trial contributed by each patient from the time of first dose to last date on trial; EAIRs were calculated as number of patients/PY × 100.

b Investigators decided whether laboratory values qualified as adverse events.

c Includes the preferred terms hypertension, hypertensive crisis, essential hypertension, labile hypertension, and systolic hypertension.

d Urinary tract infection is one of the most common non-neurological complications in patients with multiple sclerosis.^27^

e Both were cases of new-onset UC.

### TEAEs of special interest

Rates of TEAEs of special interest are shown in Table 4. EAIRs of infections and serious infections were 21.9/100 PY and 1.0/100 PY, respectively, in the pooled UC + RMS population and were similar in the UC (18.9/100 PY and 1.4/100 PY) and RMS (22.9/100 PY and 0.9/100 PY) populations. The EAIR of herpes zoster was 0.5/100 PY in the pooled UC + RMS population, with similar rates in the UC (1.0/100 PY) and RMS (0.4/100 PY) populations. Three herpes zoster events in the UC population led to treatment discontinuation. As previously reported,^9^ there was 1 case of progressive multifocal leukoencephalopathy (PML) in a 46-year-old female patient with RMS who had received ozanimod for approximately 4 years before discontinuing treatment; the patient had a nonfatal outcome with neurologic sequelae. The most frequently occurring infections and serious infections are shown in Table S3.

**Table 4:** TEAEs of special interest in the UC, RMS, and pooled UC + RMS populations.

TEAEs	UC (*n* = 1158)			RMS (*n* = 2494)		UC + RMS (*n* = 3652)	
	*n* (%)		EAIR/100 PY^a^	*n* (%)	EAIR/100 PY^a^	*n* (%)	EAIR/100 PY^a^
**Infections**	437 (37.7)	18.9		1596 (64.0)	22.9	2033 (55.7)	21.9
**Serious infections**	48 (4.1)	1.4		106 (4.3)^b^	0.9	154 (4.2)	1.0
**Herpes zoster^c^**	35 (3.0)	1.0		50 (2.0)	0.4	85 (2.3)	0.5
**Malignancies**							
**Any malignancy**	24 (2.1)	0.7		41 (1.6)	0.3	65 (1.8)	0.4
**Cutaneous malignancies**	11 (<1)	0.3		14 (<1)	0.1	25 (<1)	0.2
**Basal cell carcinoma**	8 (<1)	0.2		11 (<1)	0.1	19 (<1)	0.1
**Bowen’s disease**	1 (<1)	0.03		1 (<1)	0.01	2 (<1)	0.01
**Squamous cell carcinoma**	1 (<1)	0.03		1 (<1)	0.01	2 (<1)	0.01
**Carcinoma** in situ **of skin**	1 (<1)	0.03		0	0	1 (<1)	0.01
**Dermatofibrosarcoma protuberans**	0	0		1 (<1)	0.01	1 (<1)	0.01
**Malignant melanoma**	0	0		1 (<1)	0.01	1 (<1)	0.01
**Squamous cell carcinoma of skin**	1 (<1)	0.03		0	0	1 (<1)	0.01
**Gastrointestinal-related malignancies**							
**Colon cancer**	1 (<1)	0.03		1 (<1)	0.01	2 (<1)	0.01
**Adenocarcinoma of colon**	1 (<1)	0.03		0	0	1 (<1)	0.01
**Rectal adenocarcinoma**	1 (<1)	0.03		0	0	1 (<1)	0.01
**Rectal cancer stage II**	1 (<1)	0.03		0	0	1 (<1)	0.01
**Cardiovascular disorders**							
**Hypertension^d^**	75 (6.5)	2.3		230 (9.2)	1.9	305 (8.4)	2.0
**Bradycardia^e^**	6 (<1)	0.2		7 (<1)	0.1	13 (<1)	0.1
**Myocardial ischemia**	3 (<1)	0.1		5 (<1)	0.04	8 (<1)	0.1
**Ischemic stroke**	5 (<1)	0.1		2 (<1)	0.02	7 (<1)	0.04
**Sinus bradycardia**	3 (<1)	0.1		3 (<1)	0.02	6 (<1)	0.04
**Pulmonary embolism**	3 (<1)	0.1		3 (<1)	0.02	6 (<1)	0.04
**Deep vein thrombosis**	3 (<1)	0.1		1 (<1)	0.01	4 (<1)	0.02
**Complete AV block^f^**	1 (<1)	0.03		0	0	1 (<1)	0.01
**Macular edema^g^**	8 (<1)	0.2		5 (<1)	0.04	13 (<1)	0.1
**Drug-induced liver injury^h^**	2 (<1)	0.1		1 (<1)	0.01	3 (<1)	0.02

Abbreviations: AV, atrioventricular; EAIR, exposure-adjusted incidence rate; PY, patient-years; RMS, relapsing multiple sclerosis; TEAE, treatment-emergent adverse event; UC, ulcerative colitis.

a PY were calculated as the sum of the number of years on trial contributed by each patient from the time of first dose to last date on trial; EAIRs were calculated as number of patients/PY × 100.

b Includes 1 case of progressive multifocal leukoencephalopathy, the details of which were previously published.^9^

c Includes varicella zoster virus infection.

d Includes preferred terms hypertension, hypertensive crisis, essential hypertension, labile hypertension, and systolic hypertension.

e As determined by the investigator.

f Occurred in a 75-year-old patient with elevated body mass index and a history of long-standing hypertension, did not require ozanimod disruption, and was attributed to atherosclerotic disease affecting cardiac conduction.

g Includes macular edema events that were confirmed by a Macular Edema Review Panel.

h All 3 events reported by investigators were nonserious, did not meet Hy’s law, and did not require ozanimod disruption.

EAIRs of total malignancies and cutaneous malignancies were 0.4/100 PY and 0.2/100 PY, respectively, in the pooled UC + RMS population and were similar in the UC (0.7/100 PY and 0.3/100 PY) and RMS (0.3/100 PY and 0.1/100 PY) populations. The most common cutaneous malignancy was basal cell carcinoma, with an EAIR of 0.1/100 PY in the pooled UC + RMS population; other cutaneous malignancies were Bowen’s disease and squamous cell carcinoma in 2 patients each, and carcinoma in situ of skin, dermatofibrosarcoma protuberans, malignant melanoma, and squamous cell carcinoma of skin in 1 patient each. No cutaneous malignancies led to treatment discontinuation. There were also 4 gastrointestinal-related malignancies that occurred in the UC population (colon cancer, adenocarcinoma of colon, rectal adenocarcinoma, and rectal cancer stage II) and 1 that occurred in the RMS population (colon cancer). Both colon cancer malignancies and the rectal cancer stage II malignancy led to treatment discontinuation.

Hypertension was the most common cardiovascular TEAE, with an EAIR of 2.0/100 PY in the pooled UC + RMS population and similar rates in the UC (2.3/100 PY) and RMS (1.9/100 PY) populations. None of the hypertension events led to treatment discontinuation. Of all patients with a hypertension TEAE (*n* = 305), 1 patient (0.3%) with UC started an antihypertensive medication at or after the event. EAIRs of bradycardia and sinus bradycardia were 0.1/100 PY and 0.04/100 PY, respectively, in the pooled UC + RMS population and were similar in the UC (0.2/100 PY and 0.1/100 PY) and RMS (0.1/100 PY and 0.02/100 PY) populations. Two patients with UC and 1 patient with RMS discontinued treatment due to bradycardia. Other cardiovascular TEAEs included myocardial ischemia in 8 patients, ischemic stroke in 7 patients, pulmonary embolism in 6 patients, deep vein thrombosis in 4 patients, and complete atrioventricular block in 1 patient. Two patients with UC and 1 patient with RMS discontinued treatment due to ischemic stroke, and 1 patient with UC discontinued treatment due to pulmonary embolism.

The EAIR of macular edema confirmed by a Macular Edema Review Panel was 0.1/100 PY in the pooled UC + RMS population, with similar rates in the UC (0.2/100 PY) and RMS (0.04/100 PY) populations. Of the 8 confirmed cases of macular edema in UC, 7 led to treatment discontinuation. Of the 5 RMS patients with confirmed macular edema, 3 discontinued treatment due to macular edema and 1 had a macular edema diagnosis that occurred 1 day after the end of the trial. Of the 7 patients with UC who discontinued treatment due to macular edema, 5 had preexisting risk factors and/or comorbid conditions; 4 of the patients had events that had resolved or were resolving, but 3 did not have follow-up data regarding resolution of events (1 of whom was lost to follow-up). Of the 7 UC macular edema events, 4 were considered mild and 3 were considered moderate, but none were serious. The 1 patient with UC who did not discontinue treatment due to macular edema was later found to have had a previous diagnosis of macular edema prior to the trial that was not known at trial entry. This patient discontinued treatment for this reason, and the event resolved. Of the 3 patients with RMS who discontinued treatment due to macular edema, 2 had preexisting risk factors and/or comorbid conditions and all events resolved. Of these 3 RMS macular edema events, 2 were considered moderate and 1 was considered severe, but none were serious. The 1 patient with a macular edema diagnosis after the end of the trial did not have preexisting risk factors, but the patient had had laser surgery, a known risk factor; however, the event was considered to be resolving. The patient with RMS who did not discontinue treatment had a preexisting risk factor, and the event of macular edema did not resolve.

Drug-induced liver injury as determined by the treating investigator occurred in 3 patients, with an EAIR of 0.02/100 PY in the pooled UC + RMS population and similar rates in the UC (0.1/100 PY) and RMS (0.01/100 PY) populations. None of the 3 hepatic events met Hy’s law or led to treatment discontinuation.

### Laboratory abnormalities

#### Hepatic enzyme elevations

In the pooled UC + RMS population, 4.7%, 2.3%, 0.3%, and 0.5% of patients had elevations  ≥ 3 times the upper limit of normal (ULN) in alanine aminotransferase (ALT), aspartate aminotransferase (AST), bilirubin, and alkaline phosphatase, respectively, during OLE trials (Table 5). Elevations  ≥5 times the ULN in ALT, AST, bilirubin, and alkaline phosphatase occurred in 1.3%, 0.9%, 0%, and 0.1% of patients, respectively, in the pooled UC + RMS population. Most ALT and AST elevations were transient and resolved without treatment interruption. In the pooled UC + RMS population, treatment discontinuation due to ALT and AST elevations occurred in 11 (<1%) and 5 (<1%) patients, respectively. No serious hepatic events occurred, and there were no Hy’s law cases.

**Table 5:** Hepatic enzyme elevations in the UC, RMS, and pooled UC + RMS populations during OLE trials.

Hepatic enzyme elevations	UC	RMS	UC + RMS
**Patients with an assessment, *n***	871	2489	3360
**ALT^a^**			
** ≥3 × ULN**	68 (7.8)	91 (3.7)	159 (4.7)
** ≥5 × ULN**	22 (2.5)	20 (0.8)	42 (1.3)
**AST^b^**			
** ≥3 × ULN**	36 (4.1)	42 (1.7)	78 (2.3)
** ≥5 × ULN**	11 (1.3)	18 (0.7)	29 (0.9)
**Bilirubin**			
** ≥3 × ULN**	0	9 (0.4)	9 (0.3)
** ≥5 × ULN**	0	0	0
**ALP**			
** ≥3 × ULN**	14 (1.6)	3 (0.1)	17 (0.5)
** ≥5 × ULN**	3 (0.3)	2 (0.1)	5 (0.1)

Abbreviations: ALP, alkaline phosphatase; ALT, alanine aminotransferase; AST, aspartate aminotransferase; OLE, open-label extension; RMS, relapsing multiple sclerosis; UC, ulcerative colitis; ULN, upper limit of normal.

a A total of 45 and 5 patients had confirmed ALT ≥3 × ULN and ≥5 × ULN, respectively, defined as a repeat elevation in ≤4 weeks.

b A total of 11 and 3 patients had confirmed AST ≥3 × ULN and ≥5 × ULN, respectively, defined as a repeat elevation in ≤4 weeks.

#### ALC reductions

ALC reduction is an expected pharmacodynamic effect of ozanimod due to its mechanism of action.^13^ ALC reductions to < 200 and < 500 cells/µL occurred in 12.8% and 68.9%, respectively, of the pooled UC + RMS population during OLE trials (Table 6). Rates of ALC reductions to < 200 and < 500 cells/µL were 6.8% and 57.9%, respectively, in patients with UC and 14.8% and 72.8%, respectively, in patients with RMS. ALC < 200 cells/µL was not temporally associated with serious or opportunistic infections. Six patients with UC and 3 patients with RMS had TEAEs of lymphopenia that led to treatment discontinuation. Five patients with UC and 4 patients with RMS had TEAEs of lymphocyte count decreased that led to treatment discontinuation.

**Table 6:** ALC Reductions in the UC, RMS, and pooled UC + RMS populations during OLE trials.

Characteristics	UC	RMS	UC + RMS
**Patients with an assessment, *n***	859	2489	3348
**<200 cells/µl, *n* (%)**	58 (6.8)	369 (14.8)	427 (12.8)
**<500 cells/µl, *n* (%)**	497 (57.9)	1811 (72.8)	2308 (68.9)

Abbreviations: ALC, absolute lymphocyte count; OLE, open-label extension; RMS, relapsing multiple sclerosis; UC, ulcerative colitis.

#### Deaths

There were 5 deaths (0.4%) in patients with UC. The causes of death included 1 of each of the following: COVID-19; mucinous adenocarcinoma of unknown origin; pneumonia influenza and acute respiratory distress syndrome in a patient with a history of chronic obstructive pulmonary disease, bilateral pulmonary nodules, ischemic cardiomyopathy, and prolonged tobacco use; sudden death in a patient with history of heart failure and hypertension in the presence of acute dehydration due to UC; and adenocarcinoma of the pancreas in a former cigarette smoker.

There were 15 deaths (0.6%) in patients with RMS. The deaths included 2 that were malignancy-related (bladder cancer and disseminated cancer with unknown primary focus), 2 that were accident-related (fatal motorbike accident and multiple craniocerebral injuries), and 2 pulmonary embolisms. The deaths also included 1 of each of the following: abscess of the right lung, COVID-19, COVID-19 bilateral pneumonia, COVID-19 infection, COVID-19 pneumonia, heart failure, intracerebral hemorrhage, pneumonia, and sudden death. There were also 2 additional deaths reported after the required safety follow-up period due to malignancies (metastatic pancreatic carcinoma and glioblastoma).

## Discussion

This integrated analysis of 3652 patients with >16 000 PY of ozanimod exposure across 2 different immunological indications (UC and RMS) demonstrated that long-term treatment was generally well tolerated and confirmed the established safety profile of ozanimod in patients with moderate to severe UC or RMS. These results add to previous reports of ozanimod safety in patients with UC and RMS,^8^^,^^9^^,^^14^ with no new safety findings being observed with longer exposure to ozanimod.

Treatment discontinuations due to TEAEs were infrequent in this analysis. Rates of most TEAEs of special interest, including serious infections, malignancies, cardiovascular disorders, and macular edema, were low and similar across both indications. However, there were notable differences in EAIRs of some TEAEs between the UC and RMS populations; anemia, arthralgia, and TEAEs related to lymphocyte decreases or hepatic enzyme elevations occurred more frequently in patients with UC, whereas headache, back pain, depression, and some infections occurred more frequently in patients with RMS. It is worth acknowledging that some of these differences in specific AEs between the 2 indications may be the result of disease context and TEAE definitions in trials rather than ozanimod-related effects. For example, anemia and arthralgia are sometimes associated with UC flares (which were classified as TEAEs in the UC trials), thus possibly contributing to the higher rates of these AEs reported in UC than RMS patients.

Infections, cutaneous malignancies, and macular edema are potential risks with S1P receptor modulation.^1^^,^^5^^,^^15^ However, serious infections were uncommon in this pooled analysis at 1.0/100 PY and were lower than those reported for vedolizumab (3.7/100 PY) or anti-TNF agents (3.4/100 PY) in UC^16^ or for most therapies (3.0–3.7/100 PY) in RMS.^17^ The incidence of herpes zoster was within the estimated range in the general population of individuals >45 years old (0.12–2.5/100 PY).^18^ PML has been reported with S1P receptor modulators, with duration of therapy and age being risk factors,^19^ but the previously reported case of PML^9^ in a patient with RMS is the first and to our knowledge the only known case in a patient treated with ozanimod in clinical trials. The incidence of cutaneous malignancies was low, with most being basal cell carcinoma, which occurred within the estimated range in the general US and European populations (0.02–0.36/100 PY).^20–24^ Macular edema events confirmed by a Macular Edema Review Panel were infrequent (0.1/100 PY). Of the 13 cases of macular edema, 10 occurred in patients with risk factors and 9 resolved or were resolving according to available follow-up data. Although the risk of macular edema is low, it is recommended that clinicians obtain a baseline evaluation of the fundus, including the macula, near the start of treatment with ozanimod. Patients should be periodically evaluated while on therapy and any time there is a change in vision, particularly if they have diabetes mellitus, uveitis, or a history of retinal disease.^1^^,^^2^ The incidence of infections, malignancies, and macular edema in this analysis were similar to those in prior analyses,^8^^,^^9^^,^^14^ suggesting that risk does not increase with longer exposure to ozanimod.

Some cardiac abnormalities may also occur with S1P receptor modulators due to activity at S1P receptors on cardiomyocytes.^25^^,^^26^ Ozanimod initially acts as an agonist at S1P receptor 1, but with continued dosing, ozanimod transitions to act as a functional antagonist via S1P receptor 1 internalization.^26^ This process may result in transient cardiac conduction adverse effects (eg, decreases in heart rate and atrioventricular conduction delays) at ozanimod initiation, which are mitigated by gradual dose escalation. In this analysis of UC and RMS clinical trials that used a 7-day ozanimod dose escalation regimen, cardiac conduction TEAEs were rare and usually did not lead to treatment discontinuation.

Hepatic enzyme elevations were observed with ozanimod,^1^ but most were transient and resolved without treatment interruption; no serious hepatic events or Hy’s law cases were observed. However, it is recommended that recent liver enzyme levels should be available at ozanimod initiation and checked periodically during treatment.^1^^,^^2^ Another laboratory abnormality assessed was ALC reduction, which is an expected pharmacodynamic effect of ozanimod, with the reduced capacity of lymphocytes to egress from lymph nodes.^1^^,^^3^ While reductions to < 500 cells/µL were common in this analysis, larger reductions to < 200 cells/µL occurred in fewer patients and—importantly—were not temporally associated with serious or opportunistic infections; this lack of association could be attributed to the fact that lymphocytes are sequestered but not depleted with ozanimod. These observations were the result of frequency of ALC monitoring in ozanimod clinical trials, which may differ from those in clinical practice. It is recommended that a recent ALC measurement should be available at ozanimod initiation and monitored periodically during treatment.^1^^,^^2^ Evidence from UC and RMS clinical trials indicates that ALC returns to normal after ozanimod is discontinued, with a median time to return of 30 days and approximately 80%–90% of patients having normal ALC within 3 months.^1^

Overall, these findings indicate that TEAEs of special interest potentially associated with S1P receptor modulation are infrequent, are manageable, and often occur within the range of the general population without an increased risk with longer exposure to ozanimod. The selectivity of ozanimod may contribute to this favorable safety profile.^3–5^ Given the consistently low incidence of safety events with ozanimod in this large study across 2 different diseases, the results from this study provide further confidence in the safety of S1P receptor modulators and may enable clinicians to individualize or streamline screening requirements, shifting toward a more selective, risk-based approach.

This analysis is not without limitations. Much of the UC data and all of the RMS data were from OLE trials, which lacked control groups and blinding. The populations studied in this analysis may not fully represent the patient populations in real-world clinical settings; however, OLE trials like those included in this analysis mimic real-life clinical practice. Differences in the patient populations and trial designs of the UC and RMS trials are also a factor that should be considered when indirectly comparing safety data between the UC and RMS populations. Notwithstanding these considerations, this study was a comprehensive analysis of the long-term safety of ozanimod across multiple indications using data from clinical trials with rigorous safety monitoring. In conclusion, the findings in this integrated analysis of ozanimod clinical trials of patients with UC or RMS confirm the established safety profile of ozanimod with >16 000 PY of exposure and up to 10 years of follow-up.

## Supplementary Material

izaf319_Supplementary_Data

## Data Availability

Bristol Myers Squibb policy on data sharing may be found at https://www.bms.com/researchers-and-partners/independent-research/data-sharing-requestprocess.html. Deidentified individual patient data will not be shared.

## References

[1] Zeposia [Package Insert]. Bristol Myers Squibb; 2024.

[2] Zeposia [Summary of Product Characteristics]. Celgene Distribution B.V.; 2024.

[3] Scott FL , Clemons B, Brooks J, et al. Ozanimod (RPC1063) is a potent sphingosine-1-phosphate receptor-1 (S1P1) and receptor-5 (S1P5) agonist with autoimmune disease-modifying activity. Br J Pharmacol. 2016;173:1778-1792. 26990079 10.1111/bph.13476 PMC4867749

[4] Chaudhry BZ , Cohen JA, Conway DS. Sphingosine 1-phosphate receptor modulators for the treatment of multiple sclerosis. Neurotherapeutics. 2017;14:859-873. 28812220 10.1007/s13311-017-0565-4 PMC5722770

[5] Yang X , Yan Y, Liu S, et al. Potential adverse events associated with sphingosine-1-phosphate (S1P) receptor modulators in patients with multiple sclerosis: an analysis of the FDA Adverse Event Reporting System (FAERS) database. Front Pharmacol. 2024;15:1376494. 38846098 10.3389/fphar.2024.1376494 PMC11153721

[6] Swallow E , Patterson-Lomba O, Yin L, et al. Comparative safety and efficacy of ozanimod versus fingolimod for relapsing multiple sclerosis. J Comp Eff Res. 2020;9:275-285. 31948278 10.2217/cer-2019-0169

[7] Paul D , Swallow E, Patterson-Lomba O, et al. Comparative effectiveness and safety of ozanimod versus other oral DMTs in relapsing-remitting multiple sclerosis: a synthesis of matching-adjusted indirect comparisons. Ther Adv Neurol Disord. 2024;17:17562864241237856. 38855023 10.1177/17562864241237856 PMC11162124

[8] Danese S , Panaccione R, Abreu MT, et al. Efficacy and safety of approximately 3 years of continuous ozanimod in moderately to severely active ulcerative colitis: interim analysis of the True North open-label extension. J Crohns Colitis. 2024;18:264-274. 37651686 10.1093/ecco-jcc/jjad146 PMC10896634

[9] Cree BA , Selmaj KW, Steinman L, et al. Long-term safety and efficacy of ozanimod in relapsing multiple sclerosis: up to 5 years of follow-up in the DAYBREAK open-label extension trial. Mult Scler. 2022;28:1944-1962. 35765217 10.1177/13524585221102584 PMC9493410

[10] Sandborn WJ , Feagan BG, Wolf DC, et al.; TOUCHSTONE Study Group. Ozanimod induction and maintenance treatment for ulcerative colitis. N Engl J Med. 2016;374:1754-1762. 27144850 10.1056/NEJMoa1513248

[11] Sandborn WJ , Feagan BG, D’Haens G, et al.; True North Study Group. Ozanimod as induction and maintenance therapy for ulcerative colitis. N Engl J Med. 2021;385:1280-1291. 34587385 10.1056/NEJMoa2033617

[12] Sandborn WJ , Feagan BG, Hanauer S, et al. Long-term efficacy and safety of ozanimod in moderately to severely active ulcerative colitis: results from the open-label extension of the randomized, phase 2 TOUCHSTONE study. J Crohns Colitis. 2021;15:1120-1129. 33438008 10.1093/ecco-jcc/jjab012 PMC8256627

[13] Harris S , Tran JQ, Southworth H, et al. Effect of the sphingosine-1-phosphate receptor modulator ozanimod on leukocyte subtypes in relapsing MS. Neurol Neuroimmunol Neuroinflamm. 2020;7:e839. 32737072 10.1212/NXI.0000000000000839 PMC7413711

[14] Danese S , Cree BAC, Wolf DC, et al. Long-term safety of ozanimod in moderately to severely active ulcerative colitis and relapsing multiple sclerosis [abstract]. United Eur Gastroenterol J. 2023;11(suppl 8):437.

[15] Beaugerie L , Rahier JF, Kirchgesner J. Predicting, preventing, and managing treatment-related complications in patients with inflammatory bowel diseases. Clin Gastroenterol Hepatol. 2020;18:1324-1335.e1322. 32059920 10.1016/j.cgh.2020.02.009

[16] Karlqvist S , Sachs MC, Eriksson C, et al.; SWIBREG Study Group. Comparative risk of serious infection with vedolizumab vs anti-tumor necrosis factor in inflammatory bowel disease: results from nationwide Swedish registers. Am J Gastroenterol. 2024;119:2480-2492. 38994835 10.14309/ajg.0000000000002961 PMC11608631

[17] Knapp R , Hardtstock F, Krieger J, et al. Serious infections in patients with relapsing and progressive forms of multiple sclerosis: a German claims data study. Mult Scler Relat Disord. 2022;68:104265. 10.1016/j.msard.2022.104245 36306609

[18] Van Oorschot D , Vroling H, Bunge E, et al. A systematic literature review of herpes zoster incidence worldwide. Hum Vaccin Immunother. 2021;17:1714-1732. 33651654 10.1080/21645515.2020.1847582 PMC8115759

[19] Croteau D , Kim T, Chan V, et al. Progressive multifocal leukoencephalopathy associated with sphingosine-1-phosphate receptor ­modulators: a large case series. Mult Scler Relat Disord. 2024;92:106163. 39541823 10.1016/j.msard.2024.106163

[20] Bielsa I , Soria X, Esteve M, et al.; Skin Cancer Study Group of Barcelonès Nord. Population-based incidence of basal cell carcinoma in a Spanish Mediterranean area. Br J Dermatol. 2009;161:1341-1346. 19796178 10.1111/j.1365-2133.2009.09468.x

[21] Celić D , Lipozencić J, Jurakić Toncić R, et al. The incidence of basal cell carcinoma in Croatia: an epidemiological study. Acta Dermatovenerol Croat. 2009;17:108-112. 19595266

[22] De Vries E , Micallef R, Brewster DH, et al.; EPIDERM Group. Population-based estimates of the occurrence of multiple vs first primary basal cell carcinomas in 4 European regions. Arch Dermatol. 2012;148:347-354. 22431775 10.1001/archdermatol.2011.2244

[23] Muzic JG , Schmitt AR, Wright AC, et al. Incidence and trends of basal cell carcinoma and cutaneous squamous cell carcinoma: a population-based study in Olmsted County, Minnesota, 2000 to 2010. Mayo Clin Proc. 2017;92:890-898. 28522111 10.1016/j.mayocp.2017.02.015 PMC5535132

[24] Venables ZC , Nijsten T, Wong KF, et al. Epidemiology of basal and cutaneous squamous cell carcinoma in the U.K. 2013-15: a cohort study. Br J Dermatol. 2019;181:474-482. 30864158 10.1111/bjd.17873 PMC7379277

[25] Camm J , Hla T, Bakshi R, et al. Cardiac and vascular effects of fingolimod: mechanistic basis and clinical implications. Am Heart J. 2014;168:632-644. 25440790 10.1016/j.ahj.2014.06.028

[26] Chun J , Giovannoni G, Hunter SF. Sphingosine 1-phosphate receptor modulator therapy for multiple sclerosis: differential downstream receptor signalling and clinical profile effects. Drugs. 2021;81:207-231. 33289881 10.1007/s40265-020-01431-8 PMC7932974

[27] Medeiros Junior WLG , Demore CC, Mazaro LP, et al. Urinary tract infection in patients with multiple sclerosis: an overview. Mult Scler Relat Disord. 2020;46:102462. 32890816 10.1016/j.msard.2020.102462

