# Supplementary material for: Integrated long-term safety of 10-year ozanimod treatment: results from clinical trials in patients with moderate-to-severe ulcerative colitis or relapsing multiple sclerosis
Source: Inflamm Bowel Dis. 2026 Feb 12;32(5):954–62. doi: 10.1093/ibd/izaf319 (PMC13135834; doi:10.1093/ibd/izaf319)
Supplement: izaf319_Supplementary_Data [file izaf319_supplementary_data.zip › Rubin DT et al_supplementary data_8-26-25.docx]

**SUPPLEMENTARY DATA**

**Supplementary Table 1.** Most frequent serious TEAEs in the UC, RMS, and pooled UC + RMS populations in >4 patients in any population.

| TEAEs | UC  (N=1158) | | RMS  (N=2494) | | UC + RMS  (N=3652) | |
| --- | --- | --- | --- | --- | --- | --- |
|  | n (%) | EAIR/100 PY^a^ | n (%) | EAIR/100 PY^a^ | n (%) | EAIR/100 PY^a^ |
| UC | 54 (4.7) | 1.6 | 1 (<1) | 0.01 | 55 (1.5) | 0.3 |
| COVID-19 pneumonia | 11 (<1) | 0.3 | 21 (<1) | 0.2 | 32 (<1) | 0.2 |
| COVID-19 | 9 (<1) | 0.3 | 19 (<1) | 0.2 | 28 (<1) | 0.2 |
| Pneumonia | 6 (<1) | 0.2 | 12 (<1) | 0.1 | 18 (<1) | 0.1 |
| Appendicitis | 6 (<1) | 0.2 | 9 (<1) | 0.1 | 15 (<1) | 0.1 |
| Uterine leiomyoma | 2 (<1) | 0.1 | 12 (<1) | 0.1 | 14 (<1) | 0.1 |
| Anemia^b^ | 12 (1.0) | 0.4 | 0 | 0 | 12 (<1) | 0.1 |
| Cholelithiasis | 1 (<1) | 0.03 | 6 (<1) | 0.1 | 7 (<1) | 0.04 |
| Ischemic stroke | 5 (<1) | 0.1 | 2 (<1) | 0.02 | 7 (<1) | 0.04 |
| Epilepsy | 1 (<1) | 0.03 | 5 (<1) | 0.04 | 6 (<1) | 0.04 |
| Acute pyelonephritis | 0 | 0 | 6 (<1) | 0.1 | 6 (<1) | 0.04 |
| Spontaneous abortion | 3 (<1) | 0.1 | 3 (<1) | 0.02 | 6 (<1) | 0.04 |
| Pulmonary embolism | 3 (<1) | 0.1 | 3 (<1) | 0.02 | 6 (<1) | 0.04 |
| Hypertension^c^ | 3 (<1) | 0.1 | 3 (<1) | 0.02 | 6 (<1) | 0.04 |
| Abdominal pain | 4 (<1) | 0.1 | 1 (<1) | 0.01 | 5 (<1) | 0.03 |
| Inguinal hernia | 1 (<1) | 0.03 | 4 (<1) | 0.03 | 5 (<1) | 0.03 |
| Urinary tract infection | 3 (<1) | 0.1 | 2 (<1) | 0.02 | 5 (<1) | 0.03 |
| Intervertebral disc disorder | 0 | 0 | 5 (<1) | 0.04 | 5 (<1) | 0.03 |
| Intervertebral disc protrusion | 0 | 0 | 5 (<1) | 0.04 | 5 (<1) | 0.03 |
| Osteoarthritis | 1 (<1) | 0.03 | 4 (<1) | 0.03 | 5 (<1) | 0.03 |

^a^PY were calculated as the sum of the number of years on trial contributed by each patient from the time of first dose to last date on trial; EAIRs were calculated as number of patients/PY × 100. ^b^Investigators decided whether laboratory values qualified as adverse events. ^c^Includes preferred terms hypertension and hypertensive crisis.

Abbreviations: EAIR, exposure-adjusted incidence rate; PY, patient-years; RMS, relapsing multiple sclerosis; TEAE, treatment-emergent adverse event; UC, ulcerative colitis.

**Supplementary Table 2.** Most frequent TEAEs leading to treatment discontinuation in the UC, RMS, and pooled UC + RMS populations in >2 patients in any population.

| TEAEs | UC  (N=1158) | | RMS  (N=2494) | | UC + RMS  (N=3652) | |
| --- | --- | --- | --- | --- | --- | --- |
|  | n (%) | EAIR/100 PY^a^ | n (%) | EAIR/100 PY^a^ | n (%) | EAIR/100 PY^a^ |
| UC | 27 (2.3) | 0.8 | 0 | 0 | 27 (<1) | 0.2 |
| ALT increased^b^ | 4 (<1) | 0.1 | 7 (<1) | 0.1 | 11 (<1) | 0.1 |
| Macular edema^c^ | 7 (<1) | 0.2 | 4 (<1)^d^ | 0.03 | 11 (<1) | 0.1 |
| Lymphocyte count decreased^b^ | 5 (<1) | 0.1 | 4 (<1) | 0.03 | 9 (<1) | 0.1 |
| Lymphopenia^b^ | 6 (<1) | 0.2 | 3 (<1) | 0.02 | 9 (<1) | 0.1 |
| AST increased^b^ | 0 | 0 | 5 (<1) | 0.04 | 5 (<1) | 0.03 |
| Breast cancer | 0 | 0 | 4 (<1) | 0.03 | 4 (<1) | 0.02 |
| Liver function test increased^b^ | 3 (<1) | 0.1 | 1 (<1) | 0.01 | 4 (<1) | 0.02 |
| Anemia^b^ | 4 (<1) | 0.1 | 0 | 0 | 4 (<1) | 0.02 |
| Bradycardia | 2 (<1) | 0.1 | 1 (<1) | 0.01 | 3 (<1) | 0.02 |
| Colitis | 3 (<1) | 0.1 | 0 | 0 | 3 (<1) | 0.02 |
| Herpes zoster | 3 (<1) | 0.1 | 0 | 0 | 3 (<1) | 0.02 |
| Pneumonia | 0 | 0 | 3 (<1) | 0.02 | 3 (<1) | 0.02 |
| GGT increased^b^ | 1 (<1) | 0.03 | 2 (<1) | 0.02 | 3 (<1) | 0.02 |
| Ischemic stroke | 2 (<1) | 0.1 | 1 (<1) | 0.01 | 3 (<1) | 0.02 |
| Dyspnea | 2 (<1) | 0.1 | 1 (<1) | 0.01 | 3 (<1) | 0.02 |

^a^PY were calculated as the sum of the number of years on trial contributed by each patient from the time of first dose to last date on trial; EAIRs were calculated as number of patients/PY × 100. ^b^Investigators decided whether laboratory values qualified as adverse events. ^c^Includes macular edema events that were confirmed by a Macular Edema Review Panel. ^d^One patient received the last dose of trial drug and ended the trial 1 day before diagnosis of macular edema.

Abbreviations: ALT, alanine aminotransferase; AST, aspartate aminotransferase; EAIR, exposure-adjusted incidence rate; GGT, gamma-glutamyl transferase; PY, patient-years; RMS, relapsing multiple sclerosis; TEAE, treatment-emergent adverse event; UC, ulcerative colitis.

**Supplementary Table 3.** Most frequent infections in ≥3% of patients in any population and serious infections in >2 patients in any population in the UC, RMS, and pooled UC + RMS populations.

| TEAEs | UC  (N=1158) | | RMS  (N=2494) | | UC + RMS  (N=3652) | |
| --- | --- | --- | --- | --- | --- | --- |
|  | n (%) | EAIR/100 PY^a^ | n (%) | EAIR/100 PY^a^ | n (%) | EAIR/100 PY^a^ |
| Infections | | | | | | |
| Nasopharyngitis | 98 (8.5) | 3.0 | 531 (21.3) | 5.0 | 629 (17.2) | 4.5 |
| COVID-19 | 118 (10.2) | 3.6 | 412 (16.5) | 3.4 | 530 (14.5) | 3.4 |
| Upper respiratory tract infection | 65 (5.6) | 2.0 | 310 (12.4) | 2.7 | 375 (10.3) | 2.5 |
| Urinary tract infection | 23 (2.0) | 0.7 | 169 (6.8) | 1.4 | 192 (5.3) | 1.2 |
| Bronchitis | 31 (2.7) | 0.9 | 157 (6.3) | 1.3 | 188 (5.1) | 1.2 |
| Respiratory tract infection | 17 (1.5) | 0.5 | 165 (6.6) | 1.4 | 182 (5.0) | 1.2 |
| Viral respiratory tract infection | 23 (2.0) | 0.7 | 145 (5.8) | 1.2 | 168 (4.6) | 1.1 |
| Sinusitis | 29 (2.5) | 0.9 | 101 (4.0) | 0.8 | 130 (3.6) | 0.8 |
| Pharyngitis | 16 (1.4) | 0.5 | 107 (4.3) | 0.9 | 123 (3.4) | 0.8 |
| Influenza | 24 (2.1) | 0.7 | 84 (3.4) | 0.7 | 108 (3.0) | 0.7 |
| Rhinitis | 12 (1.0) | 0.4 | 88 (3.5) | 0.7 | 100 (2.7) | 0.6 |
| Herpes zoster^b^ | 35 (3.0) | 1.0 | 50 (2.0) | 0.4 | 85 (2.3) | 0.5 |
| Serious infections | | | | | | |
| COVID-19 pneumonia | 11 (<1) | 0.3 | 21 (<1) | 0.2 | 32 (<1) | 0.2 |
| COVID-19 | 9 (<1) | 0.3 | 19 (<1) | 0.2 | 28 (<1) | 0.2 |
| Pneumonia | 6 (<1) | 0.2 | 12 (<1) | 0.1 | 18 (<1) | 0.1 |
| Appendicitis | 6 (<1) | 0.2 | 9 (<1) | 0.1 | 15 (<1) | 0.1 |
| Acute pyelonephritis | 0 | 0 | 6 (<1) | 0.1 | 6 (<1) | 0.04 |
| Urinary tract infection | 3 (<1) | 0.1 | 2 (<1) | 0.02 | 5 (<1) | 0.03 |
| Gastroenteritis | 2 (<1) | 0.1 | 1 (<1) | 0.01 | 3 (<1) | 0.02 |
| Pyelonephritis | 1 (<1) | 0.03 | 2 (<1) | 0.02 | 3 (<1) | 0.02 |

^a^PY were calculated as the sum of the number of years on trial contributed by each patient from the time of first dose to last date on trial; EAIRs were calculated as number of patients/PY × 100. ^b^Includes varicella zoster virus infection.

Abbreviations: EAIR, exposure-adjusted incidence rate; PY, patient-years; RMS, relapsing multiple sclerosis; TEAE, treatment-emergent adverse event; UC, ulcerative colitis.

**Supplementary Figure 1.** TOUCHSTONE and True North trial designs. ^a^A 7-day dose escalation period (ozanimod 0.23 mg PO QD on days 1-4 and then ozanimod 0.46 mg PO QD on days 5-7) preceded 8 weeks of induction treatment with the assigned dose of ozanimod 0.46 mg or 0.92 mg PO QD. ^b^Patients with clinical response at Week 8 continued their blinded regimen for 24 weeks in the maintenance period. ^c^Patients without clinical response at Week 8, patients who lost response during maintenance, and patients who completed maintenance could enter the OLE. ^d^The 10-week induction period included a 7-day dose escalation period (ozanimod 0.23 mg PO QD on days 1-4 and then ozanimod 0.46 mg PO QD on days 5-7 followed by ozanimod 0.92 mg PO QD thereafter). ^e^Ozanimod-treated patients with clinical response at Week 10 were rerandomized to ozanimod or placebo for 42 weeks in the maintenance period; placebo-treated patients with clinical response at Week 10 continued to received placebo during maintenance. ^f^Patients without clinical response at Week 10, patients who lost response during maintenance, and patients who completed maintenance could enter the OLE. The TOUCHSTONE OLE was closed after all active patients completed ≥200 weeks of follow-up; those who remained in the trial at the time of closure could roll over into the True North OLE. Abbreviations: OLE, open-label extension; PO, by mouth; QD; daily.


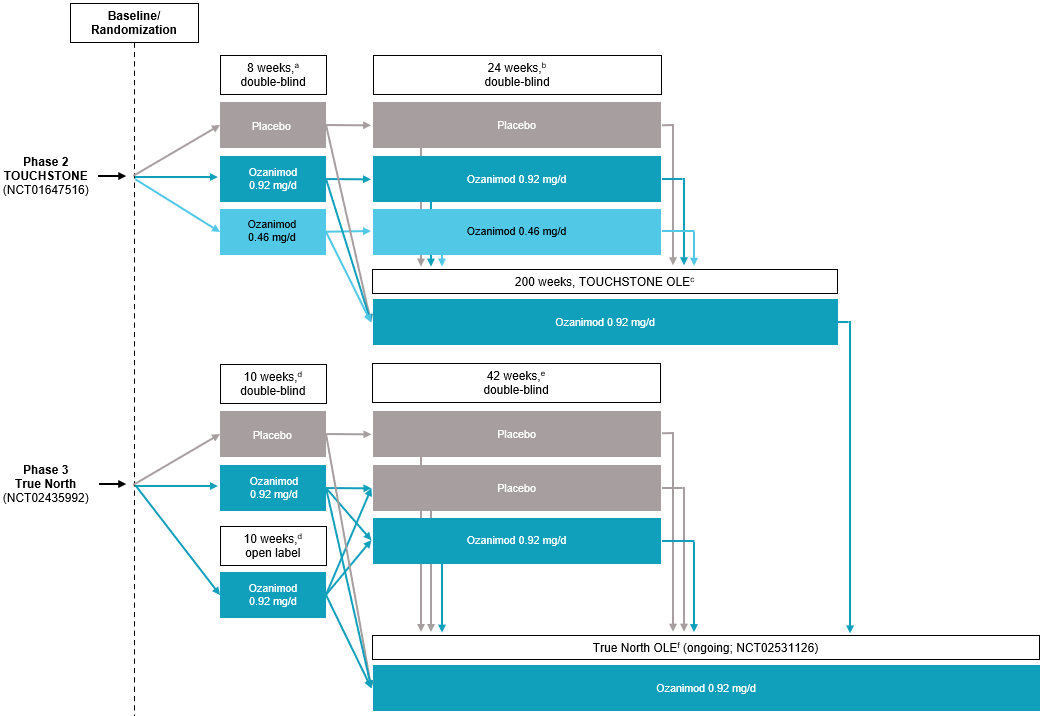


**Supplementary Figure 2.** DAYBREAK OLE trial design. Ozanimod was initiated using a 7-day dose escalation regimen (ozanimod 0.23 mg PO QD on days 1-4 and then ozanimod 0.46 mg PO QD on days 5-7 followed by the assigned dose of ozanimod 0.46 mg PO QD or 0.92 mg PO QD thereafter) in all trials. ^a^All patients entering the phase 2 dose-blinded extension period underwent dose escalation. ^b^Dose escalation was performed for all patients entering the DAYBREAK OLE from one of the phase 3 trials; dose escalation was not performed for patients entering from the phase 1 or 2 trials, unless the last dose of ozanimod was >14 days prior to entering the OLE. Abbreviations: IFN, interferon; IM, intramuscular; OLE, open-label extension; PO, by mouth; QD, daily. Adapted with permission from Selmaj KW et al. *Mult Scler Relat Disord*. 2021;51:102844 under Creative Commons Attribution 4.0 International License.

**
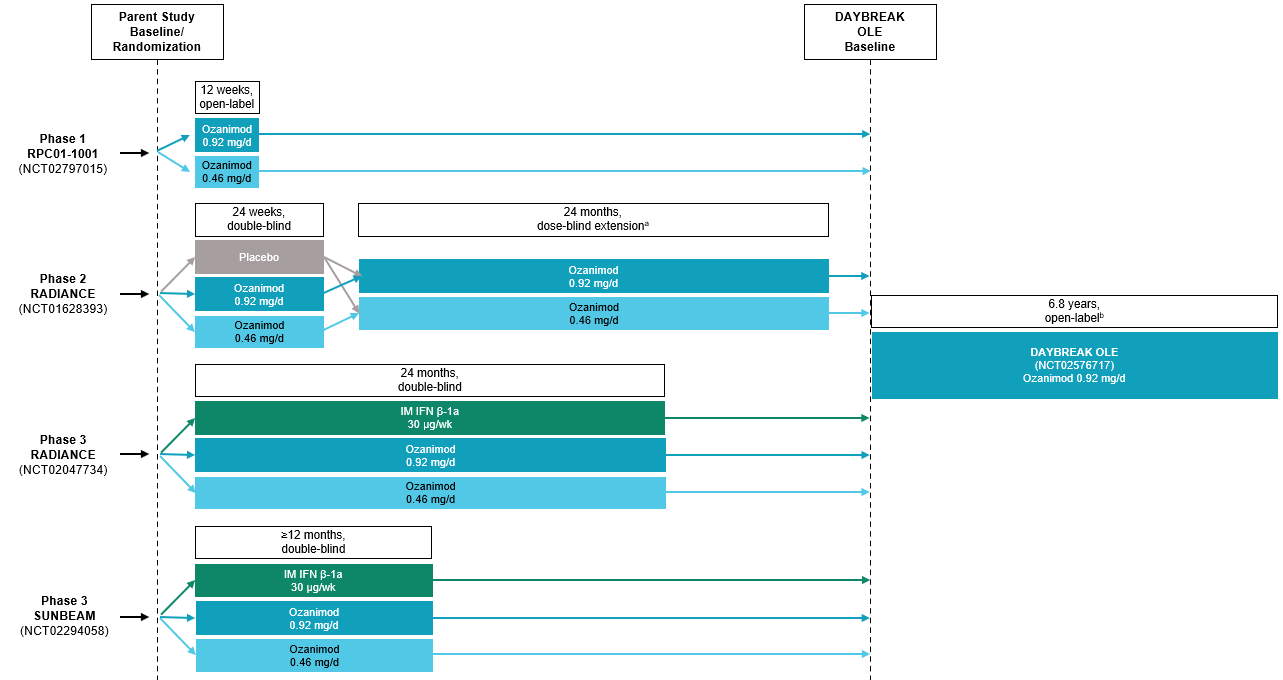
**
